# Live Attenuated *Leishmania donovani* Centrin Knock Out Parasites Generate Non-inferior Protective Immune Response in Aged Mice against Visceral Leishmaniasis

**DOI:** 10.1371/journal.pntd.0004963

**Published:** 2016-08-31

**Authors:** Parna Bhattacharya, Ranadhir Dey, Pradeep K. Dagur, Amritanshu B. Joshi, Nevien Ismail, Sreenivas Gannavaram, Alain Debrabant, Adovi D. Akue, Mark A. KuKuruga, Angamuthu Selvapandiyan, John Philip McCoy, Hira L. Nakhasi

**Affiliations:** 1 Division of Emerging and Transfusion Transmitted Disease, Center for Biologics Evaluation and Research, Food and Drug Administration, Silver Spring, Maryland, United States of America; 2 Flow Cytometry Core, National Heart, Lung, and Blood Institute, National Institutes of Health, Bethesda, Maryland, United States of America; 3 Office of Vaccines Research and Review, Center for Biologics Evaluation and Research, Silver Spring, Maryland, United States of America; 4 Institute of Molecular Medicine, New Delhi, India; Queensland Institute of Medical Research, AUSTRALIA

## Abstract

**Background:**

Visceral leishmaniasis (VL) caused by the protozoan parasite *Leishmania donovani* causes severe disease. Age appears to be critical in determining the clinical outcome of VL and at present there is no effective vaccine available against VL for any age group. Previously, we showed that genetically modified live attenuated *L*. *donovani* parasites (*LdCen-/-*) induced a strong protective innate and adaptive immune response in young mice. In this study we analyzed *LdCen-/-* parasite mediated modulation of innate and adaptive immune response in aged mice (18 months) and compared to young (2 months) mice.

**Methodology:**

Analysis of innate immune response in bone marrow derived dendritic cells (BMDCs) from both young and aged mice upon infection with *LdCen-/-* parasites, showed significant enhancement of innate effector responses, which consequently augmented CD4^+^ Th1 cell effector function compared to *LdWT* infected BMDCs *in vitro*. Similarly, parasitized splenic dendritic cells from *LdCen-/-* infected young and aged mice also revealed induction of proinflammatory cytokines (IL-12, IL-6, IFN-γ and TNF) and subsequent down regulation of anti-inflammatory cytokine (IL-10) genes compared to *LdWT* infected mice. We also evaluated *in vivo* protection of the *LdCen-/-* immunized young and aged mice against virulent *L*. *donovani* challenge. Immunization with *LdCen-/-* induced higher IgG2a antibodies, lymphoproliferative response, pro- and anti-inflammatory cytokine responses and stimulated splenocytes for heightened leishmanicidal activity associated with nitric oxide production in young and aged mice. Furthermore, upon virulent *L*. *donovani* challenge, *LdCen-/-* immunized mice from both age groups displayed multifunctional Th1-type CD4 and cytotoxic CD8 T cells correlating to a significantly reduced parasite burden in the spleen and liver compared to naïve mice. It is interesting to note that even though there was no difference in the *LdCen-/-* induced innate response in dendritic cells between aged and young mice; the adaptive response specifically in terms of T cell and B cell activation in aged animals was reduced compared to young mice which correlated with less protection in old mice compared to young mice.

**Conclusions:**

Taken together, *LdCen-/-* immunization induced a significant but diminished host protective response in aged mice after challenge with virulent *L*. *donovani* parasites compared to young mice.

## Author Summary

Visceral leishmaniasis (VL) is caused by the protozoan parasite *Leishmania donovani*. There is no effective vaccine available against VL for any age group and importantly, there are no previous studies regarding immune responses against experimental *Leishmania* vaccines tested in aged animals. We have reported earlier that immunization with a live attenuated *L*. *donovani* parasites (*LdCen-/-*) induced protective immune response in young animals viz, mice, hamsters and dogs. In this study we analyzed *LdCen-/-* mediated modulation of innate and adaptive responses in aged mice and compared to young mice. We observed that *LdCen-/-* infected dendritic cells from young and aged mice resulted in enhanced innate effector functions compared to *LdWT* parasites both *in vitro* and *in vivo*. Further, upon virulent challenge, *LdCen-/-* immunized young and aged mice displayed protective Th1 immune response which correlated with a significantly reduced parasite burden in the visceral organs compared with naïve challenged mice. Although there was no difference in the *LdCen-/-* induced dendritic cell response between aged and young mice; adaptive response in aged was reduced, compared to young which correlated with less protection in aged compared to young mice. This study supports the potential use of *LdCen-/-* as vaccine candidate across all age groups against VL.

## Introduction

Visceral leishmaniasis caused by the protozoan parasite, *Leishmania donovani*, is fatal when left untreated. Epidemiological studies show that more than 90% of VL infections are concentrated in five countries viz, India, Brazil, Bangladesh, Nepal, and Sudan [1]. According to the World Health Organization report, Leishmaniasis accounts for roughly 1 out of every 1000 deaths due to infectious disease [2] and the mortality rates are similar for both young and old adults [2]. However, other reports suggest that 85% of cases of VL occur in children [3] thereby presenting a higher prevalence of VL in this group [4, 5]. In addition, there are contradictory reports on the outcome of cutaneous leishmaniasis (CL) infection caused by *Leishmania major* (*L*. *major*) parasites in aged mice [6, 7]. This disparity in the outcome of Leishmaniasis indicates a need to better understand the modulation of immune response in aged host during *Leishmania* pathogenesis.

With increased age, the immune system declines slowly in its efficiency to fight off infectious agents which in turn results in severity of symptoms and prolonged duration of infection [8, 9]. In addition, reactivation of chronic infections occurs at a higher frequency in aged population [7]. The dysfunctions in the immune system in the aged population are mainly caused by alterations in the components of the innate and adaptive immune systems. However, in the context of the innate immune system, there are substantial evidences suggesting that innate cells, specifically APCs (macrophage, dendritic cells), maintain unaltered immune response with aging [10–13]. Nevertheless, with regard to the adaptive immune system, there is evidence for broad-ranging, age-associated deficiencies in the development and function of B and T cells [14]. Specifically, aging is associated with diminished and/or altered cytokine patterns, expression of delayed type hypersensitivity reactions to antigens encountered earlier in life, and reduction in clonal expansion of Ag-specific T and B cells [11, 15]. Importantly, the impaired ability to mount adaptive immune responses to new pathogens may result in a higher susceptibility to infectious diseases and can cause an insufficient vaccine response [16]. Indeed, reduced immune responses to vaccination have been observed for variety of vaccines including Streptococcus *pneumoniae*, influenza, hepatitis, and tetanus [17–20]. However, Tdap vaccine (‘tetanus toxoid, reduced diphtheria toxoid and acellular pertussis’) was found to be immunogenic in subjects ≥65 years, and resulted in a satisfactory but diminished protective antibody response in the elderly compared to young adults [21]. Additionally, it was demonstrated that live attenuated VZV vaccine (“zoster vaccine”) can elicit a significant increase in cell-mediated immunity to VZV in immunocompetent older adults which eventually prevents reactivation of herpes zoster partially or attenuates the severity of post-herpetic neuralgia [22–26]. The non-uniformity in host response against different vaccines suggests that the immunological mechanisms induced by vaccines in distinct age groups are not fully understood.

Over the past decades, chemotherapeutic treatment of VL with different type of drugs has shown only moderate success due to serious side effects and widespread emergence of drug resistant strains [27]. Given the insufficiencies associated with current treatments, vaccination could to be a practical alternative for an effective preventative control for the disease [28]. Previous attempts at vaccination based on killed *Leishmania* parasites or defined parasite antigens resulted in a limited protection [29, 30]. It is known that a cure from disease either due to a natural cutaneous infection, cutaneous leishmanization [31] or visceral leishmaniasis [32–34] protects the individual from reinfection. The notion that persistence of a few parasites in the body can sustain a life-long protection against leishmaniasis implies that adequately attenuated live *Leishmania* parasites can likewise yield protection [35–37]. Live-attenuated vaccines allow the host immune system to interact with a wide repertoire of antigens considered to be crucial in the development of protective immunity and more importantly cause no pathology [38]. However, a significant barrier to development and evaluation of vaccine candidates against leishmaniasis is to define the immunological correlates associated with protection both in young and old individuals. Therefore studies are needed to evaluate the efficacy of live attenuated vaccine candidates in all aged populations.

To address these questions, our laboratory has developed a *L*. *donovani* strain deleted for the centrin1 gene (*LdCen-/-)* that is essential for parasite virulence [39, 40]. *LdCen-/-* parasites have been shown to persist a maximum of 5–8 weeks in mice [40]. We have previously demonstrated safety, immunogenicity of *LdCen-/-* and protection against infection with virulent *L*. *donovani* as indicated by control of parasitemia and the induction of a protective adaptive immune response in various animal models (mice, hamster and dog) [40–43]. Of particular note, all these studies were performed in young animals. It is yet to be ascertained whether *LdCen-/-* immunization can similarly be protective in aged animals. In the present study, we report the immunogenicity profile of *LdCen-/-* parasites in aged mice (18 months old) and compared it with that in young mice (2 months old) in terms of both innate and adaptive immune response. We investigated the innate response in bone marrow derived dendritic cells (BMDCs) isolated from young and aged mice after *in vitro* infection with *LdCen*-/-. We found that *LdCen-/-* infection significantly enhanced production of pro-inflammatory response in both age groups compared to *LdWT* parasite infection. Further, *LdCen-/-* infected BMDCs derived from both young and old mice were able to promote the proliferation of OVA-specific CD4^+^T cells and induced strong Th1 type immune responses *in vitro*. Similarly, parasitized splenic dendritic cells isolated from *LdCen-/-* infected young and aged BALB/c mice after 4 days of infection displayed up-regulation of many proinflammatory cytokine genes along with generation of enhanced CD4^+^Th1 response compared to *LdWT* infected mice. *In vivo* studies further showed that immunization with *LdCen-/-* protected both young and aged mice from a challenge with virulent *L*. *donovani* parasites via generating enhanced Th1 predominant immune response along with generation of NO from restimulated splenocytes. Comparison of both innate and adaptive immune response showed that aged animals induced lower adaptive response with no change in the innate response. The reduced adaptive immune response correlated with satisfactory but reduced protection in aged mice compared to young mice upon virulent *L*. *donovani* challenge.

## Materials and Methods

### Animals and parasites

5 to 6-wk old Female BALB/c mice were obtained from the National Cancer Institute, National Institute of Health, Bethesda, MD, USA. Mice belonging to younger group were 8-wk old and mice belonging to aged group were 72-wk old. All mice were maintained in the FDA/CBER AAALAC-accredited facility under standard environmental conditions for this species. Among parasites, the wild type *L*. *donovani (LdWT)* (MHOM/SD/62/1S) maintained in golden Syrian hamsters and centrin1 gene–deleted (*LdCen-/-*) line of *L*. *donovani* (Ld1S2D) were used [39, 40]. The parasites were cultured according to the procedure previously described [39, 44]. Red fluorescent protein (RFP)-expressing *LdWT* parasites were developed using the pA2RFPhyg plasmid for integration of a RFP/ Hygromycin B resistance gene expression cassette into the parasite 18S rRNA gene locus as described previously [45]. mCherry expressing *LdCen-/-* parasites were generated using the pLEXSY-cherry-sat2 plasmid and following the company’s protocols (Jena Bioscience). The parasites were cultured according to the procedure previously described [44].

### Ethics statement

The animal protocol for this study has been approved by the Institutional Animal Care and Use Committee at the Center for Biologics Evaluation and Research, US FDA (ASP 1995#26). Further, the animal protocol is in full accordance with ‘The guide for the care and use of animals’ as descried in the US Public Health Service policy on Humane Care and Use of Laboratory Animals 2015 (http://grants.nih.gov/grants/olaw/references/phspolicylabanimals.pdf).

### Immunization and challenge studies

The young and aged mice were immunized via tail vein with 3X10^6^ stationary phase *LdCen-/-* promastigotes; 5-wk post-immunized mice were then challenged via tail vein with 10^5^ virulent *L*. *donovani* (*LdWT)* metacyclic parasites. In each study, at least 6 mice were used per group. Age matched naive mice used as controls were also similarly challenged with 10^5^ virulent *L*. *donovani* metacyclic parasites. After 10-wk of post-challenge period, parasite load was recorded from spleens of challenged mice by culturing the separated host cell preparations by limiting dilutions as previously described [40]. After 4-wk post challenge period proinflammatory cytokines, nitric oxide, antibody titers and T cell effector responses were measured.

### Multiplex cytokine ELISA

Splenocytes were plated in 24-well culture plates and stimulated with freeze-thaw *L*. *donovani* Ag (80μg/ml FTAg) in complete RPMI 1640 medium and cells were incubated at 37°C in 5% CO2, with 95% humidity. After 72h of culture, cell supernatants were collected and stored in −80°C until cytokines were analyzed using multiplex kits, MILLIPLEX Mouse Cytokine/Chemokine Magnetic Bead Panel (Millipore). The plate was read in a Luminex-100 (Luminex) system using Bio-Plex Manager software 5.0. The cytokine analysis procedure has been performed according to the manufacturer’s instructions, and the level of cytokine concentration was measured using a standard curve of each specific cytokine.

### Nitric Oxide (NO) quantification

Splenocytes or macrophages obtained from peritoneal fluid were cultured in complete RPMI 1640 medium in the presence of FTAg (80μg/ml) for 24h at 37°C. NO (nitrite/nitrate) production was determined from the supernatants of the cultures by the Griess Reaction Kit (Sigma-Aldrich) [40].

### Antibody responses

The IgG specific Ab responses were measured by conventional ELISA method. Briefly, ELISA plates were coated overnight at room temperature with FTAg (80 μg/ml). A serial dilution of the sera was carried out to determine the titer, which is defined as the inverse of the highest serum dilution factor giving an absorbance of >0.2. Titers for the Abs were determined using the following HRP-conjugated secondary Abs: Rabbit anti-mouse IgG (H+L)–HRP, Rabbit anti-mouse IgG1-HRP, Rabbit anti-mouse IgG2a-HRP (Southern Biotech; all with 1:1000 dilutions). SureBlue (KPL) was used as a peroxidase substrate. After 15 min, the reaction was stopped by the addition of 100 μl 1M H_2_SO_4_, and the absorbance was read at 450 nm.

### Carboxyfluorescein succinimidyl ester (CFSE) proliferation assay

The proliferative capacity of T cells was assessed by a CFSE dilution assay in *LdCen-/-* immunized mice before and after challenge with virulent *L*. *donovani* parasites. Age-matched naive mice served as negative controls for Ag-specific proliferation. Splenocytes from different groups of mice were isolated, incubated in 5μM CFSE (Molecular Probes/Invitrogen) for 10 min in RPMI 1640 without fetal calf serum (FCS), followed by 5 min of quenching in ice-cold RPMI 1640 plus 10% FCS, and subsequently washed thoroughly before plating in 96-well tissue culture plates at 2×10^5^ cells/well. Cells were cultured for 5 days at 37°C with 5% CO2 under stimulation with FTAg (80 μg/ml). Cells were harvested, washed, and blocked with anti-CD16/32 (5 μg/ml) for 20 min (4°C) and stained with anti-mouse CD3 allophycocyanin–eFluor 780, anti-mouse CD4 eFluor@450, anti-mouse CD8a eFluor@605NC (eBioscience, USA) (each with 1:200 dilution; 4°C) for 30 min. For analysis, single live cells (dead cells were excluded based on staining with the LIVE/DEAD Aqua dye) were gated for CFSE stained CD4^+^T cells and CD8^+^T cell and proliferation was calculated. Cells were acquired on an LSR II (BD Biosciences) equipped with 405-, 488-, 532-, and 638- nm laser lines using FACSDiva 6.1.2 software. Data were analyzed with FlowJo software version 9.1.5 (Tree Star, San Carlos, CA).

### Intracellular staining and flow cytometry

Splenocytes isolated from young and aged mice were cultured in 24-well plates in complete RPMI 1640 medium at 37°C and stimulated with FTAg (80 μg/ml). After 48h at 37°C, protein transport inhibitor (BD GolgiStop; BD Pharmingen) was added to the wells and the plate was incubated at 37°C for 6h. 6h after, cells were then blocked at 4°C with rat anti-mouse CD16/32 (5 μg/ml) from BD Pharmingen for 20 min. For surface staining, cells were then stained with anti-mouse CD3 APC-eFluor@780, anti-mouse CD4 eFluor@450, anti-mouse CD8a eFluor@605NC, anti-mouse CD44 FITC, and anti-mouse CCR7 PE-Cy5 Abs for 30 min (each with 1:200 dilution; 4°C). The cells were then stained with Live/Dead fixable aqua (Invitrogen/Molecular Probes) to stain dead cells. Cells were washed with wash buffer and fixed with the Cytofix/Cytoperm Kit (BD Biosciences) for 20 min (room temperature). Intracellular staining was done with anti-mouse IFN-γ PE-Cy7, anti-mouse TNF PerCp-eFluor@710, anti-mouse IL-2 BV711, anti-mouse IL-10 APC for 30 min (each with 1:300 dilution; 4°C). Cells were acquired on an LSRII (BD Biosciences) equipped with 405, 488, 532, and 638 laser lines using DIVA 6.1.2 software. Data were analyzed with FlowJo software version 9.1.5 (TreeStar). For analysis, first doublets were removed using width parameter; dead cells were excluded based on staining with the LIVE/DEAD Aqua dye. Lymphocytes were identified according to their light-scattering properties. CD4 and CD8 T cells were identified as CD3^+^ lymphocytes uniquely expressing either CD4 or CD8. Upon further gating, intracellular cytokines were measured in CD44^Hi^CCR7^Low^ cells. Fluorescence minus one controls were used for proper gating of positive events for designated cytokines.

### Cultivation of BMDCs

Dendritic cells were cultured *in vitro* from bone marrow progenitors. Briefly, both young and aged BALB/c mice were sacrificed and their femurs and tibias were excised, cleaned of tissue, and flushed with RPMI medium. Bone marrow was isolated by depletion of erythrocytes with ACK lysis buffer, and cultured with complete RPMI medium supplemented with 10% (v/v) fetal bovine serum (FBS) and 1% penicillin (20 U/ml)/ streptomycin (20 μg/ml) and 20 ng/mL GM-CSF (Peprotech, London, United Kingdom) and IL-4 (Peprotech) for 7 days to obtain >75% purity of CD11c^+^ DCs. For Phagocytosis and parasite clearance assay DCs were infected with parasites (5:1, parasite to DCs ratio). After incubation for 6h at 37°C in 5%CO_2,_ the cultures were washed with culture media to remove extracellular parasites, and the cultures were incubated in RPMI media for maximum of 72h. At 6, 24, 48 and 72h post-infection, cells were washed with PBS, fixed with methanol, stained with Giemsa, and intracellular parasite numbers were evaluated microscopically. For cytokine and NO measurements, DCs were infected with parasites and stimulated with LPS (1μg/ml) for 24h. Culture supernatants were collected at 24h post infection to evaluate cytokine (IL-12, TNF and IL-6) production with the use of sandwich ELISA kit (ebioscience) and nitric oxide production by the Griess assay. The assay was performed according to the manufacturer's instructions.

### Infection of mice, parasite burden estimation and parasitized splenic dendritic cell isolation

The young and aged mice were infected via tail vein with 3 X 10^6^ stationary phase red fluorescent *LdWT RFP*, or *LdCen-/-m-cherry* promastigotes. In each study, at least 6 mice were used per group. Age-matched naive mice used as control. At day 4 post infection, mice were sacrificed and parasite load was recorded from spleens of the *LdWT* and *LdCen-/-* infected mice by culturing the separated host cell preparations by limiting dilutions as previously described [40].

In a separate experiment the splenic DCs were sort selected. Single-cell suspensions were prepared from spleens, and RBCs were lysed using ACK lysing buffer. Dendritic cells were enriched using pan dendritic cell isolation kit (Miltenyi Biotec) and subsequent sort selecting of *Leishmania* (RFP/m-Cherry) infected splenic DCs and uninfected splenic DCs from different age groups of infected mice were done by high speed FACS cell sorter system (BD FACS Aria-IITM). Infected splenic DCs were sorted by gating live single enriched DCs for Cd11c+Ly6G-Cd11b-RFP/m-Cherry^+^. Uninfected splenic DCs after enrichment [Cd11c+ Ly6G-Cd11b- RFP/ m-Cherry^-^] were also sort selected.

### RT-PCR

Total RNA was extracted from the parasitized/uninfected splenic DCs using RNAqueous Microkit (AM1931) and RNA was extracted from total mouse splenocytes using PureLink RNA Mini kit (Ambion). Total RNA (400 nanograms) was reverse transcribed into cDNA using random hexamers by a high-capacity cDNA reverse transcription kit (Applied Biosytems). Gene expressions were determined by TaqManGene Expression Master Mix and premade TaqMan Gene Expression assays (Applied Biosystems) using a CFX96 Touch real-time system (Bio-Rad, Hercules, CA). The data were analyzed with CFX Manager soft-ware. The TaqMan Gene Expression Assay ID (Applied Biosystems) of different primers used was as follows: TNF, Mm00443258_m1; IL-12, Mm00434174_m1; IFN-γ, Mm01168134_m1; IL-6, Mm00446190_m1; IL-4, Mm00445259_m1; IL-10, Mm00439614_m1; and GAPDH, Mm99999915_m1. Expression values were determined by the 2^- DD Ct^ method where samples were normalized to GAPDH expression and determined relative to untreated sample.

### Antigen presentation assay

#### *In vitro* BMDC and T cell co-culture studies

BMDCs obtained from young and aged mice were pulsed with OVA peptide (2μg/ml) (323–339; Anaspec) and infected with *LdWT* or *LdCen-/-* for 24h. CD4^+^T cells were purified from spleens of young and aged DO11.10 transgenic mice (mice carrying this MHC class II restricted rearranged T cell receptor transgene on a H2d background react to ovalbumin peptide antigen) incubated with 5μM CFSE (Molecular Probes/ Invitrogen) for 10 min in RPMI 1640 without FCS followed by 5 min of quenching in ice-cold RPMI1640 plus 10% FCS and then washed thoroughly before plating in 96 well tissue culture plates along with OVA pulsed BMDCs. Cells were cultured for 5 days at 37°C with 5% CO_2_ and T cell proliferation was then estimated by flow cytometry by gating on CD4^+^ cells. Culture supernatants were collected at day 5 to evaluate cytokines (IFN-γ, IL-10) by ELISA using a Sandwich ELISA kit (e-bioscience). The assay was performed as per the detailed instructions of the manufacturer.

#### *Ex vivo* DCs and T cell co-culture studies

Parasitized splenic DCs obtained from both age groups of mice 4 days post infection were pulsed with OVA peptide (2μg/ml) followed by an *in vitro* co-culture experiment with OVA-specific CD4^+^T cells isolated and purified from DO11.10 transgenic young and aged mice. After 5 days of incubation at 37°C in a 5% CO_2_ humidified chamber, T cell proliferation was measured by studying the dilution of CFSE in CD4 stained T cells via Flow cytometry. The cytokine production (IFN-γ, IL-10) was assessed by flow cytometry by intracellular staining with anti-mouse IFN-γ PE-Cy7 and anti-mouse IL-10 APC antibodies.

### Statistical analysis

Statistical analysis of differences between means of experimental groups was determined by unpaired two-tailed Student t test, using Graph Pad Prism 5.0 software. A p value <0.05 was considered significant, and a p value <0.005 was considered highly significant.

## Results

### 1. *LdCen-/-* infection of dendritic cell from young and aged mice exhibits comparable protective innate immune responses both *in vitro* and *in vivo*

**Fig 1:**
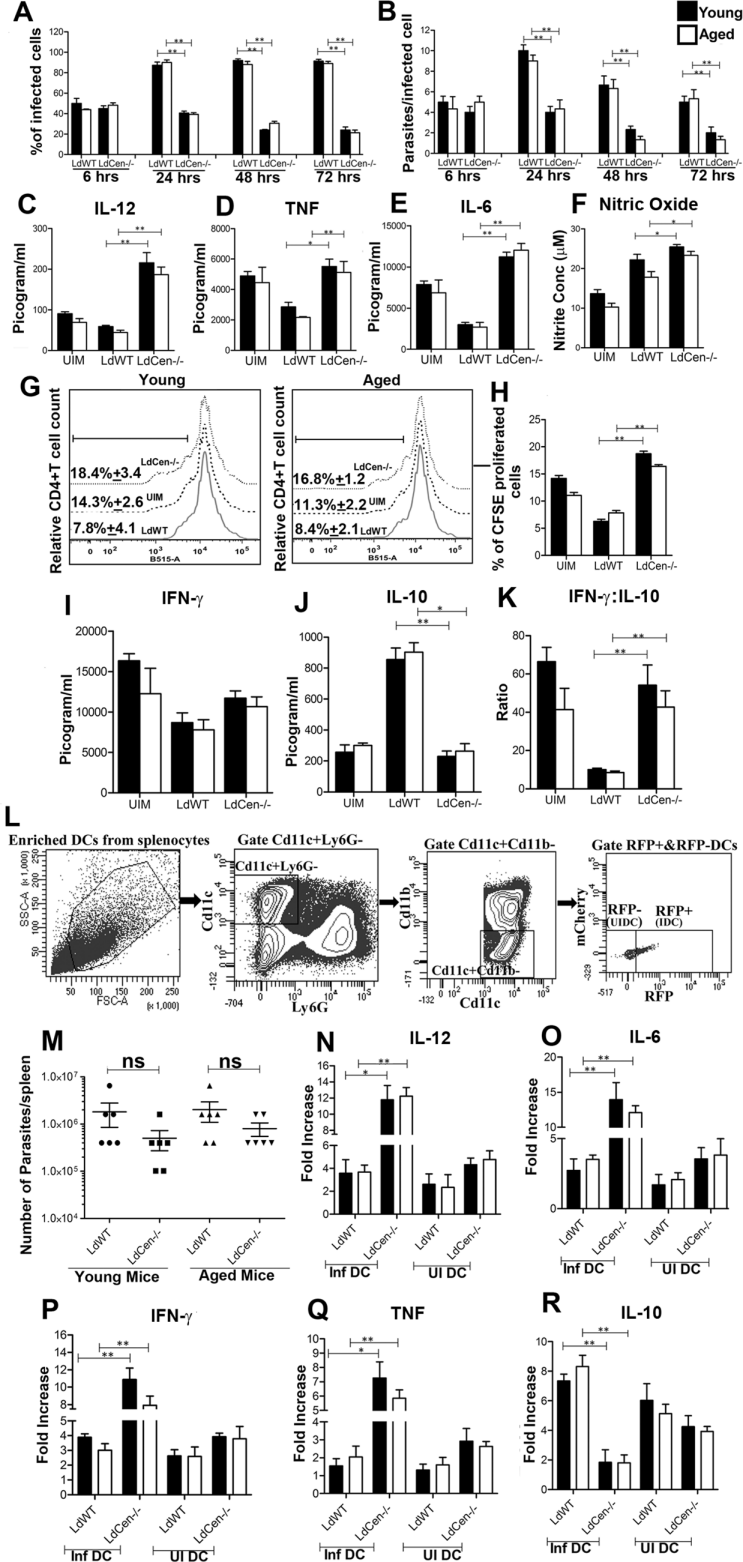
Analysis of young and aged mice derived dendritic cell function upon *LdWT* and *LdCen-/-* infection *in vitro*. BMDCs isolated from young and aged groups of mice were infected with *LdWT* or *LdCen-/-* stationary-phase promastigotes (5:1, parasite to DCs ratio) and intracellular parasite numbers were visualized by Giemsa staining and estimated microscopically at 6, 24, 48 and 72h post-infection. (A) Infection efficiency (% of infected cells) and (B) intracellular growth (parasites per infected cell) were recorded. To measure parasite load in these cultures, a minimum of 300 BMDCs were counted. In a separate experiment BMDCs were infected with parasites and stimulated with LPS (1 μg/ml) for 24h. Culture supernatants were collected to analyze IL-12 (C), TNF (D) IL-6 (E) production by ELISA and NO (F) production by the Griess assay. The data presented are representative of two independent experiments. T cell proliferation and cytokine production upon coculture of parasite-infected BMDCs with OVA-specific transgenic T cells. BMDCs obtained from young and aged BALB/c mice were pulsed with OVA peptide and were either left uninfected or infected with *LdWT* or *LdCen-/-* for 24h. CD4^+^ T cells were purified from age matched young and aged DO11.10 transgenic mice, stained with CFSE and co-cultured with parasite infected BMDCs for 5 days. (G) T cell proliferation was estimated by flow cytometry by studying CFSE dilution of gated CD4^+^ T cells and represented by the histogram. The staggered histogram overlay displays CD4^+^T cell proliferation pattern as visualized by CFSE dilution by flow cytometry. Cell proliferation was done in triplicates and histograms representative of mean values were overlaid for figure. The black line on histogram over lay represents % proliferated cells gated in CD4^+^ T lymphocytes. (H) The bar diagram representing the quantitative CFSE cell proliferation. (I, J) Culture supernatants were collected at day 5 of coculture to assay IFN-γ and IL-10 by ELISA. (K) IFN-γ: IL-10 ratio was determined. The data represent the mean values ± SD of results from 3 independent experiments that all yielded similar results. *p<0.*05*, **p<0.*005*. Following enrichment, uninfected splenic DCs (UI DC)/ bystander DCs were sorted from the spleens of different groups of infected mice (*n* = 6) by gating live single cells for [Cd11c+Ly6G-Cd11b-RFP/m-Cherry^-^] whereas parasitized splenic DCs (Inf DC) was sorted by gating live single cells for [Cd11c+Ly6G-Cd11b-RFP/m-Cherry^+^]. Sorting strategy for RFP+ and RFP- DCs is displayed (L). mCherry+ and mCherry- DCs were also sorted using the similar gating strategy. Experiment was repeated 3 times with pooled digest from 6 spleens per experiment. Parasite number in spleen of different groups of infected mice was measured 4 days post-infection (M). Mean and SEM of six mice in each group is shown. The data represent the mean values ± SD of results from 3 independent experiments that all yielded similar results. Sort selected parasitized and uninfected DCs from different groups of young and aged mice were subjected to RNA isolation as mentioned in material and methods section. Isolated total RNA was reverse transcribed and expression levels of different genes were analyzed as described in Material Methods. Normalized expression levels of proinflammatory cytokines IL-12 (N), IL-6 (O), IFN-γ (P) and TNF (Q) and anti-inflammatory cytokine IL-10 (R) were estimated. The data represent the mean values ± SD of results from 3 independent experiments that all yielded similar results.*p<0.*05*, **p<0.*005*. Black bar indicates young mice and white bar indicates aged mice. ns = not significant.

Our previous studies have shown that BMDCs from young mice manifest heightened effector function in response to *LdCen-/-* infection *in vitro* [46]. Hence, in this study we compared the ability of *LdCen-/-* parasites to modulate DC responses in BMDCs *in vitro* from both young and aged mice. *LdWT* and *LdCen-/-* parasites showed similar rates of infection along with identical parasite loads at 6h post-infection in both young and aged BMDCs (Fig 1A and 1B). These BMDCs cultures were then subsequently examined at 24, 48 and 72h post-infection, and the percentage of infected DCs and parasite loads were calculated. Interestingly, BMDCs from both young and aged mice infected with *LdCen-/-* displayed significantly lower percentage of infected cells (Fig 1A) and a lower number of parasites per infected cell (Fig 1B), compared to those infected with *LdWT* at all the time points post-infection. This indicates that *LdCen-/-* parasites infect both young and aged BMDCs similarly but are unable to persist for prolonged periods compared to *LdWT* parasites. DCs play a crucial role in coordinating immune response in leishmaniasis by providing the IL-12 necessary for the induction of protective Th1 immune response [47, 48]. *LdCen-/-* parasite infected young and aged BMDCs produced significantly more IL-12 than did those infected with *LdWT* parasites (Fig 1C). Next, we analyzed the level of proinflammatory cytokines (TNF, IL-6) and nitric oxide (NO), the key components in *Leishmania* control, in BMDCs cell supernatants 24h post infection. Interestingly, *LdCen-/-* infected BMDCs from both age groups secreted significantly more TNF, IL-6 and NO compared to *LdWT* infected cells (Fig 1D, 1E and 1F). Altogether, these observations suggest that BMDCs from both young and aged mice undergo a more pronounced effector response following infection with *LdCen-/-* compared to *LdWT* infected BMDCs. Importantly, BMDCs from aged mice showed similar innate response like that of BMDCs of young mice in response to *LdCen-/-* infection.

Next, we compared the antigen presentation capability of *LdCen-/-* infected BMDCs by measuring their ability to present *LdCen-/-* antigens and activate CFSE-labeled naïve CD4^+^ T cells as measured by IFN-γ and IL-10 production. BMDCs from young and aged mice were pulsed with OVA peptide followed by infection with *LdWT* or *LdCen-/-* and then co-cultured with OVA specific (DO11.10) CFSE labeled CD4^+^T cells. CD4^+^T cell proliferation was evaluated after 5 days by flow cytometry. *LdCen-/-* infection induced the proliferation of T cells in both young and aged BMDCs and was significantly higher compared to those co-cultured with *LdWT* infected BMDCs as demonstrated by the histogram overlay depicting the individual % proliferated CD4^+^T cells from different sets (Fig 1G) and the subsequent quantitative bar diagram (Fig 1H). Interestingly, *LdCen-/-* mediated induction of CD4^+^T cell proliferation was similar in young and aged BMDCs thereby highlighting the similar dendritic cell activation. Cytokine production resulting from CD4^+^T cell response was measured in culture supernatants after 5 days of co-culture. IFNγ levels from *LdWT* and *LdCen-/-* infected BMDCs–T cell co-cultures did not differ significantly between the two age groups and were comparatively higher in *LdCen-/-* infected BMDCs (Fig 1I). On the other hand, *LdWT* infected BMDCs–T cell co-cultures significantly induced IL-10 production compared to *LdCen-/-* infected BMDCs-T cell co-cultures in both age groups (Fig 1J). The IFN-γ: IL-10 ratio was significantly higher in *LdCen-/-* infected BMDCs-T cell cultures compared to *LdWT* infected BMDCs-T cells co-cultures (Fig 1K). Thus *in vitro* results suggest that *LdCen-/-* infected BMDCs from both age groups are capable of inducing the proliferation of Th1 type of CD4^+^T cells in *vitro* that may lead to enhanced adaptive immune response and also there is no diminution of *LdCen-/-* induced innate response between two age groups.

Further, we analyzed *LdCen-/-* mediated modulation of the dendritic cell (DCs) response in spleen using an *in vivo* mouse model. We infected young and aged mice intravenously with red fluorescent *LdWT* RFP, or *LdCen-/-* m-Cherry parasites. After 4 days we enriched DCs from spleen and sort selected uninfected DCs (UI DC) by gating live single cells for Cd11c+Ly6G-Cd11b-RFP/m-Cherry^-^ whereas parasitized DCs (Inf DC) were sorted by gating live single cells for Cd11c+Ly6G-Cd11b-RFP/m-Cherry^+^ (Fig 1L). We observed, *LdCen-/-* infected young and aged mice had comparatively lower although not statistically significant level of splenic parasite burden compared to *LdWT* infected mice (Fig 1M) which is in agreement with our *in vitro* observation. We also assessed the expression of proinflammatory and anti-inflammatory cytokine gene expression in *LdCen-/-* infected DC population and compared them with *LdWT* parasitized DCs. RT-PCR analysis showed that 4 major proinflammatory cytokines such as IL-12 (Fig 1N), IL-6 (Fig 1O), IFN-γ (Fig 1P) and TNF (Fig 1Q) were significantly elevated in parasitized splenic DCs isolated from *LdCen-/-* infected young and aged mice compared to *LdWT* infected mice. In contrast, anti-inflammatory cytokine IL-10 (Fig 1R) expression was significantly reduced in parasitized DCs isolated from *LdCen-/-* infected young and aged mice compared to *LdWT* infected mice. We did not see any significant difference in the behavior between infected DCs (Inf DC) and bystander DCs (UI DC) from *LdWT* infected young and aged mice. However, we observed that bystander DCs from *LdCen-/-* infected mice resulted in comparatively lower production of proinflammatory cytokines and higher production of anti-inflammatory cytokines compared to infected DCs from both age groups (Fig 1N–1R). Moreover, un-infected splenic DCs (UI DC) from *LdCen-/-* infected mice showed slightly higher but not statistically significant level of proinflammatory cytokines (IL-12, IL-6, IFN-γ and TNF) along with down regulation of IL-10, compared to uninfected DCs from *LdWT* infected young and aged mice (Fig 1N–1R).

Additionally, we have investigated the antigen presentation capability of dendritic cells sort selected from young and aged mice which were infected with *LdWT* or *LdCen-/-* parasites for 4 days. We pulsed these DCs with OVA peptide followed by co-culture with OVA-specific CD4^+^T cells from both age groups for 5 days to analyze OVA-specific proliferation of T cells. Consistent with our *in vitro* study, the percentage of proliferating CD4^+^T cells after 5 days were comparatively higher upon co-culture with OVA-pulsed DCs from *LdCen-/-* infected young and aged mice compared to *LdWT* infected mice (S1B Fig). Cytokine production resulting from such proliferated CD4^+^T cell response was measured by flow cytometry. The results showed that DCs from *LdCen-/-* parasite infected young and aged mice stimulated CD4^+^ T cells to produce significantly higher levels of IFN-γ/IL-10 ratio compared to CD4^+^T cells cocultured with DCs from *LdWT* infected mice (S1C Fig), thereby indicating a similar Th1 response predominance in both age groups of mice. Thus, from the *in vivo* gene expression profile and antigen presentation assay, it is also evident that *LdCen-/-* infection results in similar and significant enhancement of innate effector responses in young and aged mice.

### 2. *LdCen-/-* immunization protects both young and aged mice against challenge with virulent *L*. *donovani*

**Fig 2:**
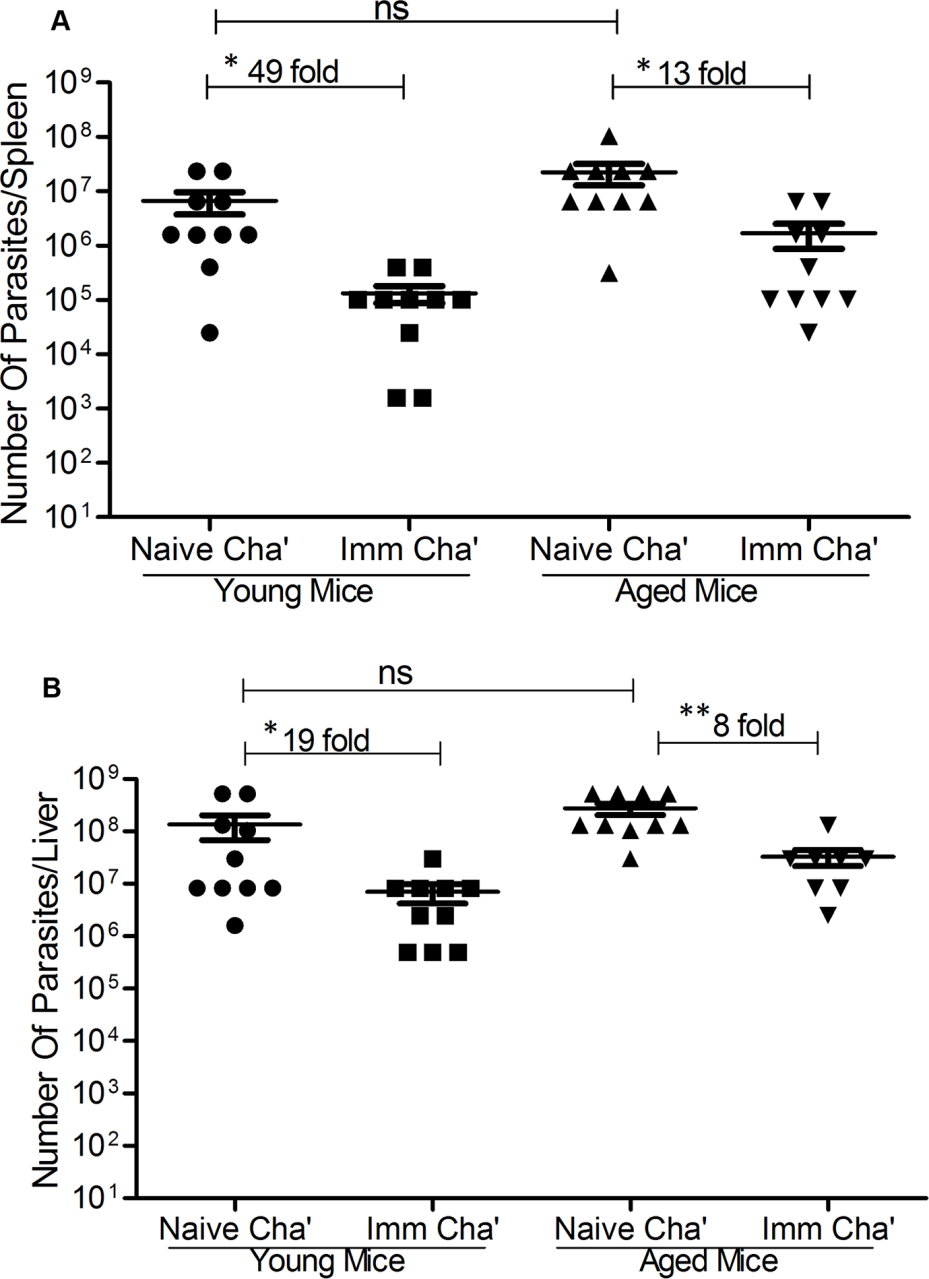
Immunization with *LdCen-/-* protects young and aged mice against virulent *L*. *donovani* challenge. Young and aged mice were immunized either with *LdCen-/-* or with PBS. 5-wk post-immunized mice with *LdCen-/-* were challenged with virulent *L*. *donovani* parasites. Parasite numbers in spleen (A) and liver (B) of young and aged mice were measured 10-wk post-challenge. The data presented are representative of three independent experiments with similar results. Mean and SEM of six mice in each group are shown. *p<0.*05*, **p < 0.*005*. ns = not significant.

The ability of *LdCen-/-* to protect against virulent *L*.*donovani* infection was determined in 5-wk immunized mice followed by 10-wk of challenge with virulent *L*. *donovani* parasites. The results showed that immunization with *LdCen-/-* significantly reduced spleen and liver parasite burden in both young and aged mice compared to naive-challenged mice (Fig 2A and 2B). The parasite burden in spleen and liver of naive challenged aged mice was similar like that of naïve challenged young mice (Fig 2A and 2B). However, the reduction in parasite burden was significantly less in the *LdCen-/-* immunized–challenged aged group (13 fold in spleen and 8 fold in liver) than the immunized–challenged young mice (49 fold in spleen and 19 fold in liver; data in Fig 2A and 2B). Overall, these data suggest that *LdCen-/-* immunization confers substantial protection in young and aged mice against virulent *L*. *donovani* challenge; however, the protection is reduced in aged mice.

### 3. *LdCen-/-* immunization induces a mixed pro-inflammatory and anti-inflammatory cytokine response in young and aged mice upon virulent *L*. *donovani* challenge

**Fig 3:**
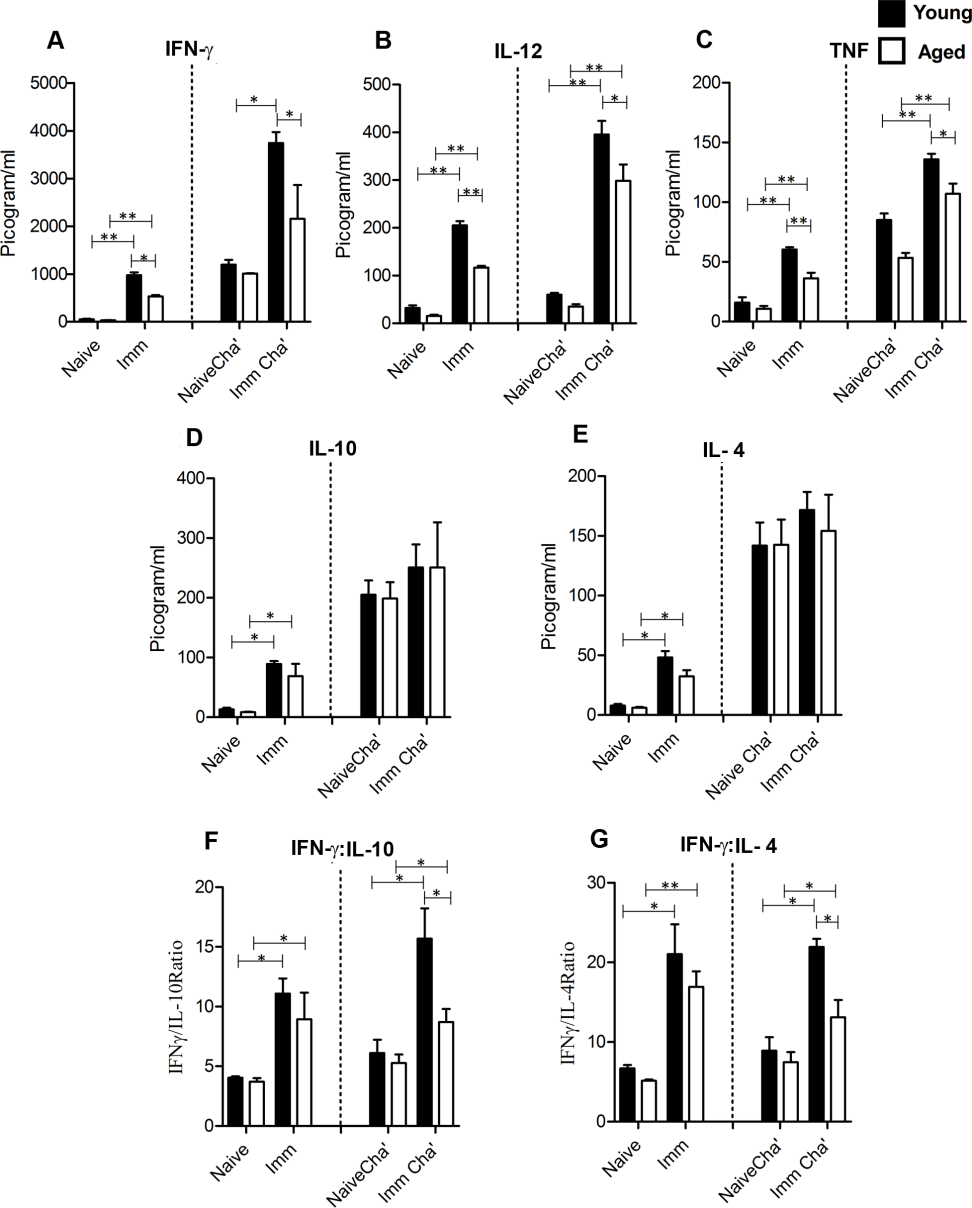
*Leishmania* Ag–stimulated cytokine profiles in splenocytes culture supernatants from naive, *LdCen-/-* immunized (Imm), naive challenged (Naive Chal), and *LdCen-/-* immunized challenged (Imm Chal) young and aged mice. The 5-wk post immunized mice were challenged with virulent *L*. *donovani* for 4-wk and then mice were euthanized, splenocytes were isolated, plated aseptically (2×10^5^ cells/well), and stimulated with *Leishmania* FTAg for 72h. Concentrations of pro-inflammatory cytokines IFN-γ (A), IL-12 (B) and TNF (C) anti-inflammatory cytokines IL-10 (D) and IL-4 (E) were measured in culture supernatants using the multiplex mouse cytokine kit as described in the Material and Methods section. Ratio of IFN-γ/IL-10 (F) and IFN-γ/IL-4 (G) were also determined. The data presented are representative of two independent experiments with similar results (n = 6). Mean and SEM of each group are shown. *p<0.*05*, **p < 0.*005*. Black bar indicates young mice and white bar indicates aged mice.

Effective clearance of *Leishmania* parasites requires a proinflammatory response which is mainly mediated by IFN-γ [49–53]. In order to evaluate the protective immunity induced after *LdCen-/-* immunization in both young and aged mice, we analyzed Ag-specific cytokine secretion by splenocytes from naive, immunized (5-wk), non-immunized challenged (naïve challenged) and immunized challenged mice (5-wk immunization + 4-wk of challenge) (Fig 3). Comparative cytokine profiles demonstrated a significant induction of *Leishmania* Ag–specific IFN-γ, IL-12, and TNF secretion in the culture supernatants of 5-wk post-immunized young and aged mice compared to naïve mice at protein level (Fig 3A–3C). Additionally, the steady state cytokine gene expression pattern at mRNA level (S2A–S2C Fig) was similar with Ag stimulated cytokine secretion at protein level. Interestingly, following challenge with virulent *L*. *donovani* parasites, the immunized young mice showed significantly enhanced IFN-γ, IL-12 and TNF secretion at both protein (Fig 3A–3C) and mRNA level (S2A–S2C Fig), while in aged mice significantly enhanced secretion of IL-12, TNF but not IFN-γ was observed (Figs 3A–3C and S2A–S2C). Importantly, overall induction of all the proinflammatory cytokines (IFN-γ, IL-12 and TNF) was significantly lower in aged mice compared to young mice from all the groups at both protein (Fig 3A–3C) and mRNA level (S2A–S2C Fig). Additionally, in immunized young and aged mice, compared with naive mice, significant induction of anti-inflammatory cytokines like IL-10 and IL-4 was observed at protein level (Fig 3D and 3E) whereas at mRNA level comparatively higher although statistically non-significant levels of IL-10 and IL-4 were observed (S2D and S2E Fig). However, young and aged immunized-challenged mice showed slightly higher although not statistically significant levels of IL-10 and IL-4, compared to naïve challenged groups at both the protein (Fig 3D and 3E) and mRNA levels (S2D and S2E Fig). Importantly, the IFN-γ: IL-10 or IFN-γ: IL-4 ratios were higher in immunized as well as immunized challenged young and aged mice compared to naïve and non-immunized challenged groups respectively (Fig 3F and 3G). Interestingly IFN-γ: IL-10 or IFN-γ: IL-4 ratios in immunized challenged aged mice were significantly less compared to young mice. Taken together, these results suggest that *LdCen-/-* immunization initiates mixed pro- and anti-inflammatory immune response against virulent *L*. *donovani* challenge in both young and aged mice albeit lower proinflammatory response in aged mice.

### 4. Immunization with *LdCen-/-* induced robust NO production and a strong anti-leishmania antibody response in young and aged mice

**Fig 4:**
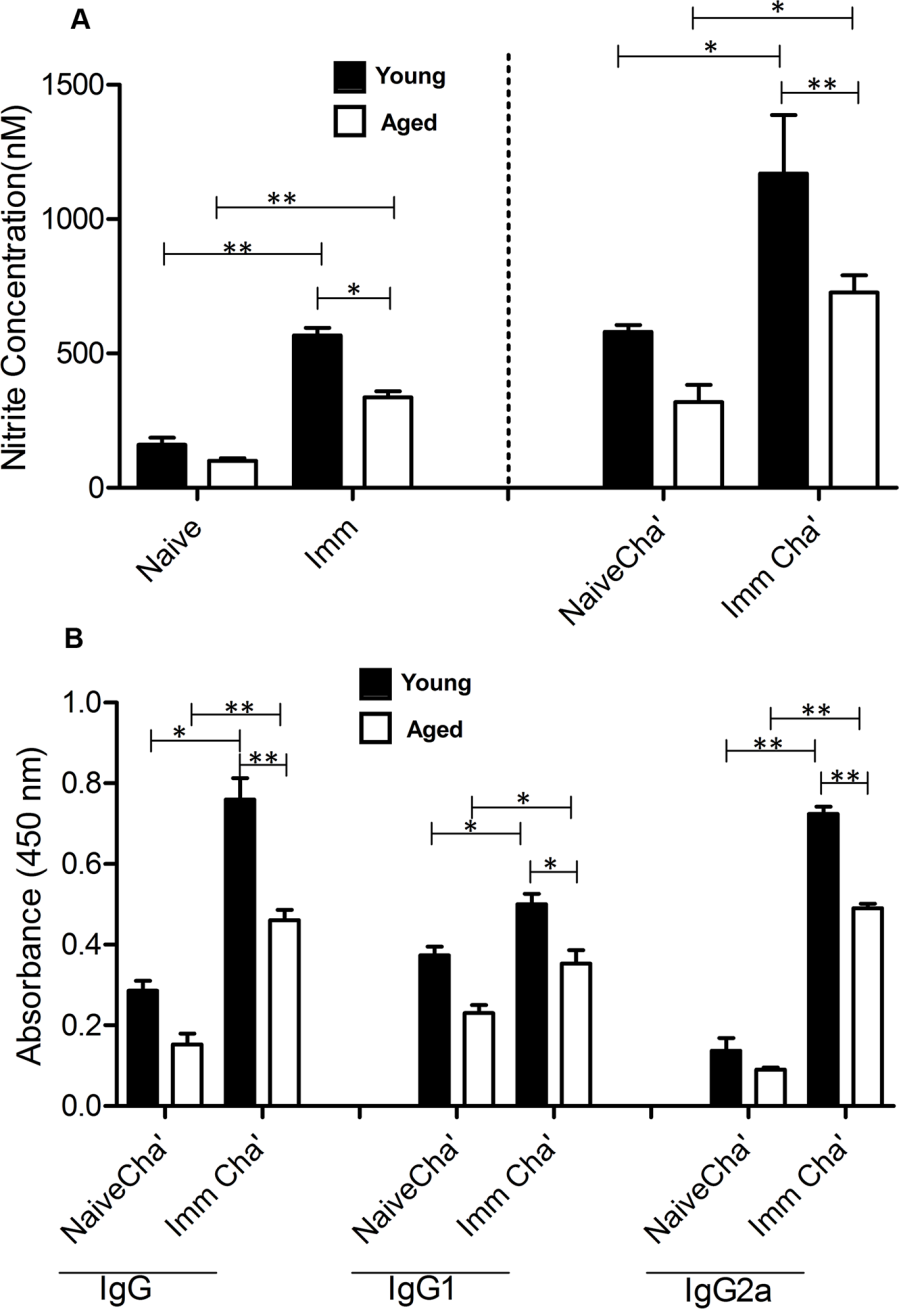
*LdCen-/-* immunization induces NO production by splenocytes along with IgG2a antibody response from the serum of virulent *L*. *donovani* challenged young and aged mice. *Leishmania* Ag specific stimulation of NO synthase (NOS_2_) in the splenocytes of naive, *LdCen-/-*immunized, naive challenged and *LdCen-/-* immunized challenged. The activity of NOS_2_, indicated by the amount of released nitrite (NO) in the Ag-stimulated splenocytes supernatants (24h) was measured by the Griess reaction (A). ELISA measurement of IgG, IgG1, and IgG2a Abs in sera from the mice immunized with *LdCen-/-* parasites for 5-wk or naïve at 4-wk post challenge (B). The data presented are representative of two independent experiments with similar results. Mean and SEM of six mice in each group are shown. *p < 0.*05*, **p < 0.*005*. Black bar indicates young mice and white bar indicates aged mice.

Since the preferential production of proinflammatory cytokines specifically IFN-γ results in increased synthesis of host protective nitric oxide (NO) by activated macrophages [54, 55], we determined the level of NO produced by *Leishmania* Ag restimulated splenocytes derived from the *LdCen-/-* immunized young and aged mice before and after challenge. It is important to note that NO product observed in the splenocytes is mainly from the macrophages present in such cultures. A significant amount of *Leishmania* Ag specific nitrite production was observed in both young and aged post immunized mice compared with non-immunized mice splenocytes (Fig 4A). Moreover, a much greater amount of NO was observed in *LdCen-/-* immunized young and aged mice upon virulent *L*. *donovani* challenge than in naive challenged mice (Fig 4A). Of note, the induction of nitrite concentration was significantly lower in aged mice compared to young mice from all the groups. Additionally, since, higher level of anti-leishmania antibody (IgG2a) dictates predominant Th1response [56, 57]; we analyzed sera from immunized-challenged young and aged mice for determination of *Leishmania*–specific IgG, IgG1, and IgG2a titers. The *Leishmania* Ag-specific IgG_Total_, IgG1, and IgG2a production was significantly higher in immune challenged young and aged mice compared to naïve challenged groups (Fig 4B). Of note, specific increase in Th1 mediated IgG2a titers in immunized and challenged mice sera from both groups were observed. Importantly, the levels of all three Ab populations were reduced in aged mice compared to young mice from all the groups.

### 5. Robust Leishmania Ag specific lymphoproliferative responses are maintained in both aged and young mice after *LdCen-/-* immunization

**Fig 5:**
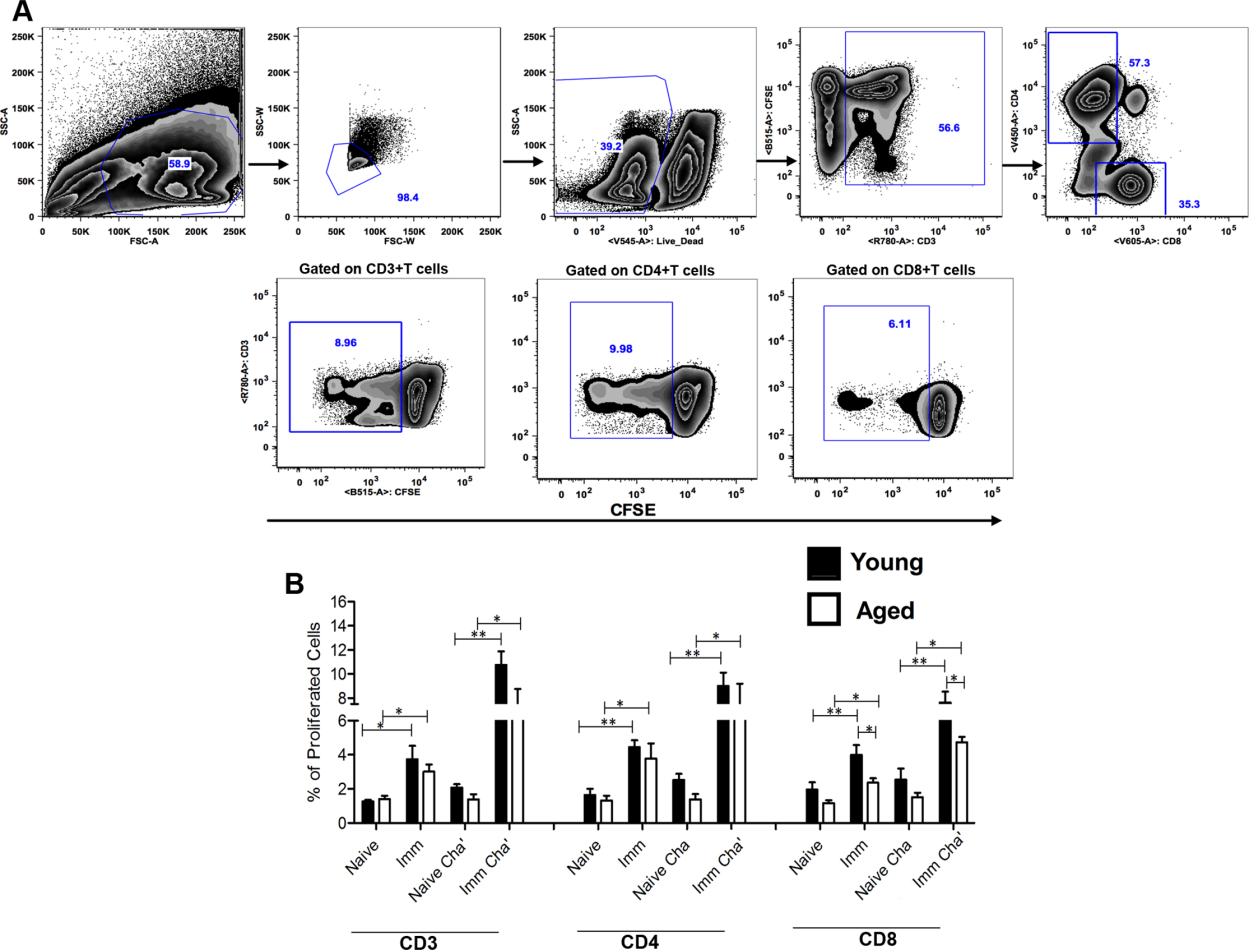
Proliferative capacity of Ag-specific T cells in young and aged mice after 8-wk post- immunization with *LdCen-/-*. Splenocytes were isolated from each naive, 8-wk post immunized (Imm), naive and 4-wk post challenged (Naive Chal), 8-wk post immunized and 4-wk post challenged (Imm Chal) mouse; stained with CFSE, and stimulated *in vitro* with FTAg for 5 days. (A) Cells were analyzed by flow cytometry, in which CFSE dilution on particular gated cells was used as the readout for Ag-specific proliferation. (B) Percentages of gated CD3, CD4, and CD8-proliferated T cells were calculated. The data presented are representative of two independent experiments with similar results and six mice in each group. Mean and SEM of each group were shown. *p < 0.*05*, **p < 0.*005*. Black bar indicates young mice and white bar indicates aged mice.

Aging is known to be associated with a decline in T cell proliferation [11, 58]. Hence to investigate if *LdCen-/-* vaccination induces *Leishmania* Ag specific T cell proliferation in young and aged mice, we performed proliferation assays using splenocytes from naïve, 8-wk post immunized, naive challenged (4-wk of challenge) and immunized challenged (8-wk immunization + 4-wk of challenge) mice that were stimulated with *Leishmania* FTAg. Results showed that both CD4 and CD8 T cells isolated from both young and aged mice immunized with *LdCen-/-*, have significantly higher proliferative capacity in response to *Leishmania* FTAg compared to T cells isolated from naive mice (Fig 5B). Further, T cells isolated from mice immunized and challenged with virulent *L*. *donovani* parasites have significantly higher proliferative capacity than naive challenged group. The significant proliferative capacity of T cells even in 8-wk post-immunized young and aged mice suggests that some of them may have derived from a memory T cell response since by that time the majority of mice have cleared the *LdCen-/-* parasite [40]. Interestingly significantly lower proliferative capacity of only CD8 T cells was observed in the spleen of immunized aged mice compared to young mice before and after challenge.

### 6. Induction of multifunctional Th1 effector cells correlates with *LdCen-/-* induced protective immunity in young and aged mice

**Fig 6:**
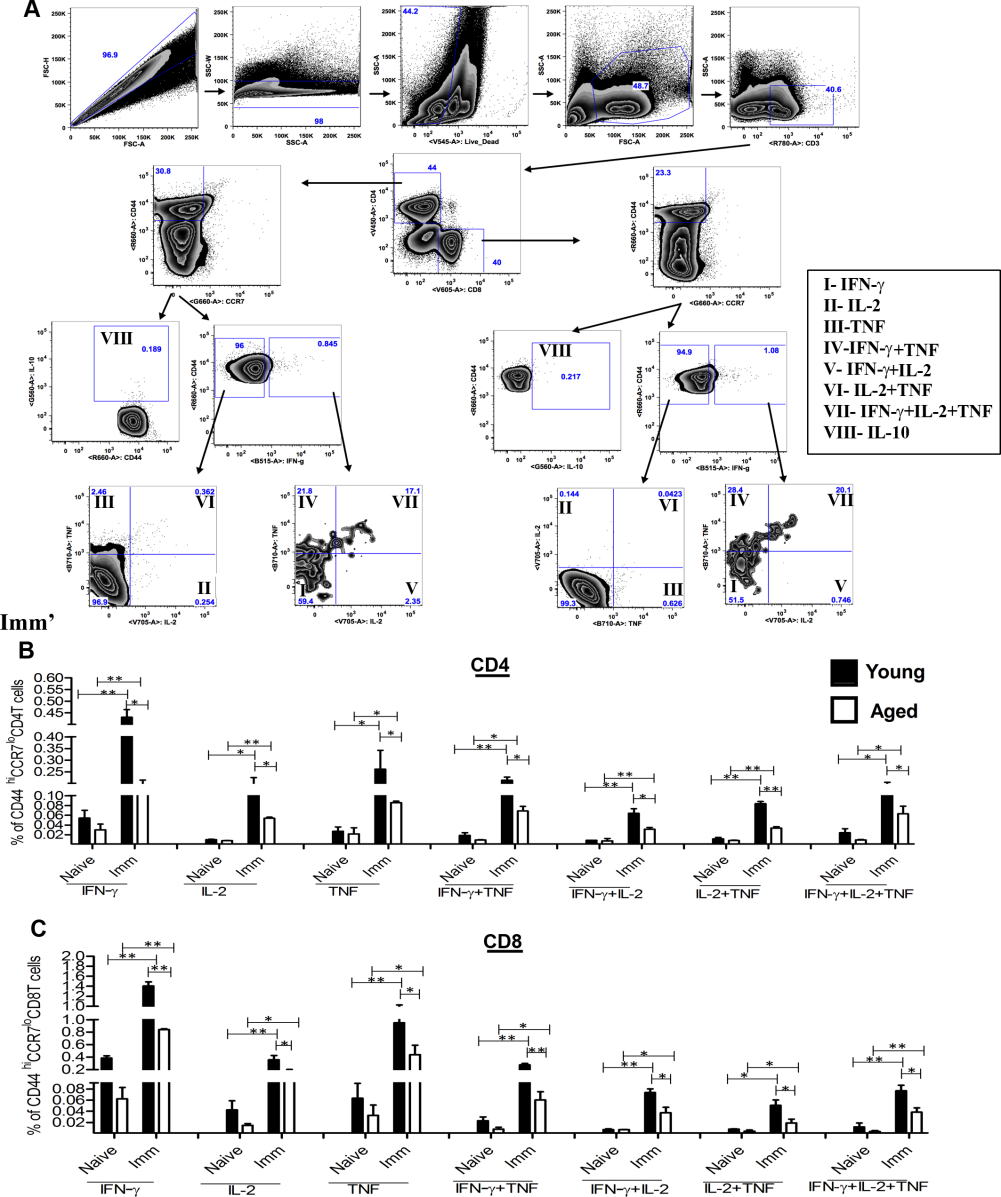
Multiparameter flow cytometry based analysis for single, double, or triple cytokine–secreting CD44^Hi^/CCR7^Low^/ CD4 or CD8 T cells from *LdCen-/-* immunized and non-immunized young and aged mice. (A) The common gating steps shown in this study. Spleen cells of 8-wk post immunized mice were stimulated with FTAg for 48h and stained with various Abs as described in Materials and Methods. Ag-experienced effector cells were gated and divided into seven distinct subpopulations, and the frequencies of the various subpopulations were calculated. (B) Cytokine analysis of CD4 T cells from naïve and 8-wk post-immunized mice. (**C**) Cytokine analysis of CD8 T cells from naïve and 8-wk post-immunized mice. The data presented are representative of two experiments with similar results. Mean and SEM of six in each group is shown. *p < 0.*05*, **p < 0.*005*. Black bar indicates young mice and white bar indicates aged mice.

**Fig 7:**
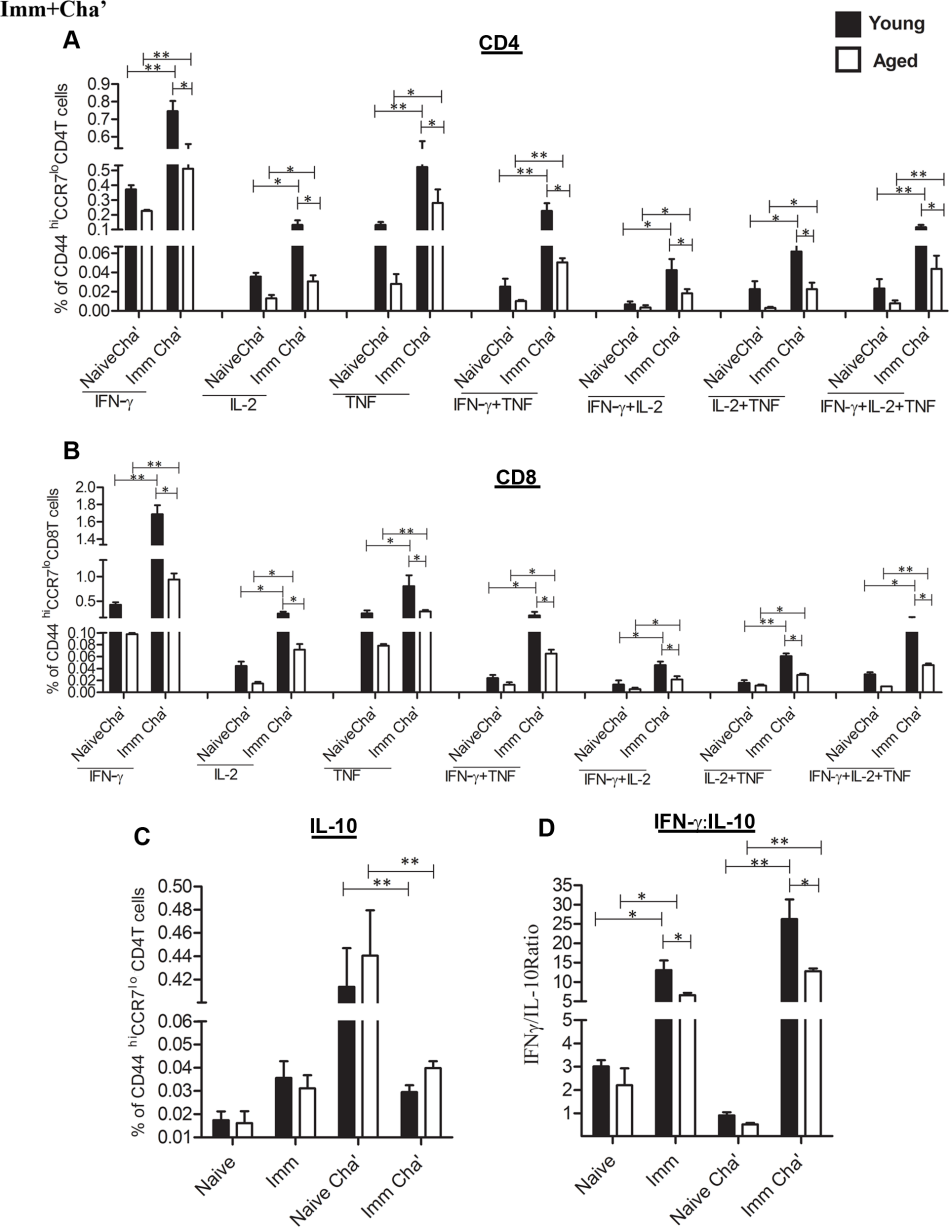
Ag-specific intracellular cytokine secretion analysis of CD4 and CD8 T cells from *LdCen-/-* immunized and non-immunized young and aged mice after virulent *L*. *donovani* challenge. The 8- wk post-immunized or non-immunized young and aged mice were challenged for 4-wk with virulent *L*. *donovani*. Intracellular cytokine analysis was done as shown in Fig 6A and divided into seven distinct subpopulations. (A) Cytokine analysis of CD4 T cells from 8-wk post immunized and 4-wk post challenged mice. (B) Cytokine analysis of CD8 T cells of 8-wk post immunized and 4-wk post challenged mice. (C) IL-10 secreting CD4 T cells and (D) the ratio of IFN-γ to IL-10 producing CD4 T cells from spleens at the time of challenge [(naive and immunized (8W)] and after challenge [(naive-challenged and immune-challenged (8WI plus 4WPC)]. The data presented are representative of two experiments with similar results. Mean and SEM of six mice in each group are shown. *p < 0.*05*, **p < 0.*005*. Black bar indicates young mice and white bar indicates aged mice.

Since efficacy of several experimental *Leishmania* vaccine candidates has been shown to correlate with the T cells secreting multiple pro-inflammatory cytokines [59], we investigated whether *LdCen-/-* immunization could induce splenic CD4^+^ and CD8^+^ T cells to secrete multiple cytokines such as IFN-γ, IL-2 and TNF in infected aged mice as was previously observed in young mice [40]. Specifically, we demonstrated cytokine production from the antigen experienced effector memory T cells (CD44^Hi^/CCR7^Low^) in 8-wk post-immunized young and aged animals. Spleen cells cultured *in vitro* in presence of *Leishmania* FTAg followed by multicolor staining were gated according to their surface expression of CD44 and CCR7. Seven distinct populations of cytokine-producing cells were defined from the CD44^Hi^/CCR7^Low^ cells based on different combinations of IFN-γ, IL-2 or TNF (Fig 6A). The results showed that at 8-wk post-immunization in both young and old mice, significantly higher number of CD4 and CD8 T cells were single, (IFN-γ, IL-2 and TNF) double (IFN-γ^+^ TNF^+^, IFN-γ^+^IL-2^+^, TNF^+^IL-2^+^) and triple cytokine producing cells (IFN-γ^+^ TNF^+^ IL-2^+^) (Fig 6B and 6C) compared to naïve mice. However, the number of single and multiple cytokine producing cells were significantly lower in aged mice compared to young mice. Similar analysis conducted with spleen cells of immunized and challenged young and aged mice showed significantly higher percent of the single and multiple cytokine-producing CD4 and CD8 T cells compared to naive challenged mice (Fig 7A and 7B). Again we observed a reduced percent of single and multiple cytokine expressing CD4 and CD8 T cell in aged immunized challenged mice.

We also quantified Ag-experienced CD4 T cells that produce IL-10, a crucial anti-inflammatory cytokine in the pathogenesis of VL from the spleen of young and aged mice (Fig 7C). Ag-experienced CD4 T cells from *LdCen-/-* immunized and challenged mice produced significantly lower IL-10 in both age groups compared to naïve challenged mice (Fig 7C). Since the balance between pro and anti-inflammatory cytokines predicts the outcome of protective immune response, we measured IFN-γ/IL-10 ratio. In Ag-stimulated CD4 T cells, the IFN-γ/IL-10 ratio was significantly higher in the immunized mice both before challenge and after challenge compared with either naive or naive-challenged controls (Fig 7D). However in aged mice the IFN-γ/IL-10 ratio was significantly lower compared to young mice. In summary, *LdCen-/-* immunized young and aged mice induced a strong antigen experienced effector T cell memory response after 8-wk post-immunization, at a time point when the majority of mice have cleared the *LdCen-/-* parasite. However, the effector T cell mediated immune response is lower in aged mice.

## Discussion

Immune dysfunction is a hallmark of aging leading to an increased susceptibility to infectious diseases in older individuals [16, 60]. In an older adult, the benefits of vaccination to prevent infectious disease are limited, mainly because of the adaptive immune system’s inability to generate protective immunity [16]. This has been observed for a variety of vaccines including *Streptococcus pneumoniae*, influenza, hepatitis, and tetanus and is thought to be the result of a compromised immune response [17–20]. However, on the other hand Tdap vaccine (‘tetanus toxoid, reduced diphtheria toxoid and acellular pertussis’) gives a satisfactory but diminished protective response in the elderly (≥65 years of age) compared with that in young adults [21]. Additionally, live attenuated zoster vaccine renders effective but partial protection against herpes zoster and postherpetic neuralgia in immunocompetent persons 60 years of age and older via boosting cell-mediated immunity to varicella–zoster virus (VZV) [22]. These differences in the efficacy of vaccines in elderly population suggest that there is a need to validate the efficacy of each vaccine in aged population. As a first step, it is important to analyze the essential components of both innate and adaptive immunity in aged and compare with the young to find out the essential differences between the two, which can be exploited to enhance the immunity of vaccines for aged against various infectious diseases including Leishmaniasis.

Till date no licensed *Leishmania* vaccines exist for any age groups. Our laboratory has shown, using genetically modified *LdCen-/-* parasites, the induction of host protective innate [61], adaptive immune response and long-term protection against virulent *L*. *donovani* infection in younger animals (mice, hamsters and dogs) [40–43]. Since, there are no reports regarding the immuno-protective role of experimental *Leishmania* vaccines including live attenuated parasites in aged animals, in this report we have performed a comprehensive study to systematically analyze the preclinical efficacy of *LdCen-/-* parasites *vis-à-vis* through induction of innate and adaptive immune response in aged mice and to compare with young mice against virulent *LdWT* infection.

The dendritic cell (DC) mediated activation of innate immune response plays a critical role in initiating and shaping Th1-protective responses [62]. Activation of DCs converts them into fully functional APCs capable of priming T cell responses [63]. Substantial evidence suggests that DCs preserve immune responses with aging [10, 11, 64, 65]. Notably, DCs obtained from elderly persons is reported to be able to present antigen as well as DCs from young donors [65]. Moreover, peripheral blood dendritic cells re-induce proliferation or clonal expansion in *in vitro* aged T cell populations thereby postponing the clonal elimination of antigen-specific T cell populations [66]. These studies suggest that at least a subset of APC in the elderly retain optimal function [67]. Previously we had shown that *LdCen-/-* modulates immune responses in young mice by acting on dendritic cells [46]. We therefore assessed whether BMDCs phagocytic function, *LdCen-/-* mediated APC function and DC-T cell interaction is impacted in aged mice *in vitro*. We found that *LdCen-/-* parasites were phagocytized at similar rate to *LdWT* parasites and did not persist for longer time in both young and aged BMDCs. Further, we observed both young and aged mice derived BMDCs infected with *LdCen-/-* parasites produced significantly more IL-12, necessary for the induction of protective Th1 response [47], compared with those infected with *LdWT* parasite-infected BMDCs which may likely induce heightened Th1 response in both age groups of mice. In addition, enhanced secretion of TNF and NO, directly involved in the killing of intracellular parasites [68], in *LdCen-/-* infected BMDCs compared with *LdWT* infection clearly indicated that *LdCen-/-* parasites not only have impaired growth inside DCs, but they also induce a proinflammatory response in both age groups, suggesting that *LdCen*-/- parasites are safe and immunogenic in both age groups. Additionally, OVA-pulsed BMDCs from young and aged mice infected with *LdCen-/-* manifest similar level of enhanced antigen presenting function as indicated by OVA specific proliferation of T cells compared to those infected with *LdWT*. Further such an interaction between BMDCs and T cells *in vitro* also resulted in enhanced CD4^+^Th1 cell activation as evident by higher IFN-γ and reduced IL-10 release by the responding T cells compared to *LdWT* infected BMDCs. Taken together, these data indicate that *LdCen-/-* infection in DCs substantially boost the CD4^+^Th1 cell effector functioning *in vitro* in both age groups. Additionally, the *in vitro* observations were further confirmed by *in vivo* experiments. We observed, the parasitized splenic DCs from *LdCen-/-* infected young and aged mice at 4 days post-infection exhibited similar and significant up regulation of proinflammatory cytokine genes such as IL-12, IL-6, IFN-γ and TNF whereas down regulation of anti-inflammatory cytokine gene IL-10 compared to *LdWT* infected mice. Consistent with our *in vitro* observation, dendritic cells sort selected from *LdCen-/-* parasitized young and aged mice 4 days post infection, resulted in the generation of similar level of enhanced CD4^+^Th1 response in young and aged mice as indicated by the higher IFN-γ/IL10 ratio compared to *LdWT* infected mice. Thus, both *in vitro* and *in vivo* observation suggest that *LdCen-/-* infection induced innate effector response in young and aged mice and interestingly, there is no diminution of *LdCen-/-* induced innate response in aged mice. Further, these findings are in agreement with studies done with TLR agonists showing myeloid DCs manifest preserved TLR-mediated immune responses with aging [10]. Additionally, it was also shown that TLR ligand-treated DCs can enhance the otherwise defective response of aged naive CD4 T cells via the induction of inflammatory cytokine specifically IL-6 [69]. Likewise, we found *LdCen-/-* substantially induces the innate effector function of aged BMDCs via production of proinflammatory molecules such as TNF, IL-6 which could likely improve residual functions of ‘‘defective” aged naive CD4^+^T cells thereby generating good adaptive response against virulent *L*. *donovani* challenge.

With regards to adaptive immunity, there have been conflicting reports about the tendency of senescent mice to mount either a Th1 or a Th2-like cytokine response [70]. Some studies report a bias for a Th2 response in aging mice [71], while others demonstrate a predominance of murine T cells releasing IFN-γ [72, 73]. A recent study has also shown a, unique reversal to a Th1 response along with attenuation of Th2 cell response in senescent mice during *L*. *major* infection leading to the development of resistance against cutaneous leishmaniasis [6]. Effective clearance of *Leishmania* parasites requires Th1 cells to secrete a substantial amount of IFN-γ [53], whereas susceptibility to *Leishmania* parasites is mainly mediated by Th2 cytokines, such as IL-10. Consistent with that finding, in our current study we observed *LdCen-/-* immunization induced significantly enhanced secretion of proinflammatory cytokine like IFN-γ over IL-10 or IL-4 in the spleen cells of immunized as well as immunized-challenged young and aged mice compared to naïve controls thereby suggesting a protective role of *LdCen-/-* in mediating the shift from Th2 to Th1 response in *L*. *donovani* challenged mice, along with possible activation of IFN-γ producing cells. Thus similar like our *in vitro* observation, *LdCen-/-* immunization in aged mice also induces a predominant proinflammatory microenvironment *in vivo* in terms of higher production of IFN-γ, IL-12 and TNF at both protein and mRNA level that may improve the residual functions of aged naive CD4^+^T cells. These observations are consistent with a study where introducing a combination of the inflammatory cytokines markedly enhanced the effector responses of the aged CD4 T cells *in vivo* [74]. Therefore, *LdCen-/-* immunization induced heightened proinflammatory response in aged mice further corroborates with protection in this group.

Proinflammatory (Th1) cytokine induced NO production is the main leishmanicidal mechanism of murine macrophages [55] and in this context, we also observed augmented NO generation in *LdCen-/-* immunized young and aged mice before and after challenge compared to naïve control. Additionally, we also detected a robust Th1-specific serum Ab (IgG2a) response in the immune-challenged young and aged mice, further highlighting *LdCen-/-* induced a generalized Th1 type response in both age groups and might also contribute to pathogen clearance [56, 57]. Strikingly, remarkable increase in the level of NO and IgG2a titers related to *LdCen-/-* immunized aged mice when compared to naïve group would indicate heightened proinflammatory response and further reinforce the protection induced by immunization with *LdCen-/-*. Of note, when compared to young mice, the induction of nitrite concentration and IgG2a titer level was significantly lower in aged mice from all the groups which is in agreement with other studies [15, 75] and corroborate with the diminished protection in aged mice. Indeed, it has been shown that the attenuated response to immunization in elderly individuals vaccinated against tetanus, is associated with decreased numbers of specific Ab- secreting B cells and usually decreased potency of those B cells [20]. Nevertheless, it has been reported that age-related reductions in humoral responses are mainly due to defects in the cognate helper function of naive CD4^+^ T cells from aged individuals [15]. This was further substantiated with hepatitis B vaccination, where it was shown that the defect in antibody production for the majority of non-responding old people results from a T-cell dysfunction [19].

T cell responses are also critical in determining the outcome of infections with *Leishmania* [49]. Notably, T cell proliferation in response to *Leishmania* antigens is an important biomarker of immunogenicity of a vaccine in mice [76]. Consistent with these reports, our results showed that vaccination with *LdCen-/-* parasites induces a higher T cell proliferation (both CD4 and CD8) upon stimulation with *Leishmania* antigen in both young and aged mice which corroborates with protection in both groups. The increase in *Leishmania* specific T cell proliferation in the immunocompromised aged mice after immunization with live attenuated parasite, is in agreement with earlier studies in human that found live attenuated varicella-zoster virus (VZV) vaccine enhanced frequency of VZV-specific proliferating T cells in PBMC of elderly vaccines [24]. Of particular note, we observed significant decrease in the proliferative capacity of CD8 T cell but not CD4 T cells in immunized and immunized challenged aged mice compared to young mice. Notably, since CD8 T cells contribute to the reduction in parasite burden during *Leishmania donovani* infection through cytotoxic activity [77] suggesting that the lower proliferation of CD8 T cells might be the underlining cause of diminished protection observed in aged mice. Indeed, several studies have shown age-related decline in overall T cell functions and its impact on vaccine efficacy and immunity [58, 78]. For example, immunization of young adults with live vaccine against influenza virus provides 65–80% protection compared to 30–50% protection in the elderly. The poor responsiveness to influenza vaccination has been shown to be specifically associated with the presence of high proportions of a population of inactive CD8^+^T lymphocytes that lack expression of the co-stimulatory molecule CD28 [58, 78, 79]. Additionally, in order to compare further the correlates of immune protection for *LdCen-/-* parasites between young and aged mice, we analyzed Ag-experienced effector memory T cells. We observed, the level of Ag-specific T cell recall response was significant, as indicated by T cell proliferation even after 8-wk post-immunization in the absence of parasite persistence, suggesting the generation of Ag-specific memory cells in both age groups and further corroborates with protection in both groups against virulent challenge. Nevertheless, compared to young mice the effector memory T cell mediated immune response is lower in aged mice. Overall, our results are in accordance with other studies showing that decline in T cell proliferation is associated with cellular senescence [11, 58] but that response is significant enough to provide protection against *Leishmania* infection in aged albeit lower than in young mice.

In order to determine the effector function of proliferated T cells, we further assessed the production of intracytoplasmic cytokines after immunization in young and aged mice. Apart from CD4^+^T cells, CD8^+^T cells play a critical role in the control of *L*. *donovani* infection by contributing to the formation of granulomas in the liver of *L*. *donovani* infected mice [77, 80]. Therefore, we analyzed both CD4 and CD8 T cells that are CD44^Hi^/CCR7^Low^ populations and represent Ag-experienced effector memory response to determine correlates of immune protection for *LdCen-/-* parasites in young and aged mice. Our *ex vivo* analysis showed that *LdCen-/-* immunized young and aged mice had an increased percent of *Leishmania* specific CD4^+^ and/or CD8^+^ T cells expressing Th1 cytokines (IFN-γ, TNF, and IL-2) either singly or in multiple combinations. It is worth mentioning here that the development of cell mediated immune responses capable of controlling *L*. *donovani* infection and resolving disease are critically dependent upon IFN-γ and TNF [49]. Additionally, multifunctional cytokine-producing cells are found to be associated with vaccine induced protection as reported by several other studies including ours [40, 59, 81]. Of note, the percent of Th1 cytokine secreting CD4^+^ and/or CD8^+^ T cell population increases after immunizations in both young and old mice, although the percent increase in aged mice is significantly lower than in young thereby further underscoring the reduced protection in the aged mice. Indeed, in the context of T cells, it is well documented that age-related changes in T cells, specifically CD4^+^T cells adversely impact the outcome of a humoral response contributing to reduced vaccine efficacy [11, 82]. Naive CD4^+^T cells from aged animals produce about half the IL-2 as young cells and resulting in reduced differentiation of effector populations that produce reduced amounts of effector cytokines [58, 74]. Additionally, age-related decline in CD4^+^T cell helper activity results in the generation of defective CD8^+^T cell responses in terms of memory response [11]. Interestingly, we found a significantly increased IFNγ/IL-10 ratio in CD4T cells in immunized-challenged mice compared with non-immunized challenged mice, suggesting that *LdCen-/-* altered the Th1/Th2 balance towards a protective Th1 type response which correlated with protection in both age groups of mice. However, comparatively lower IFNγ/IL-10 ratio in immunized challenged aged mice compared to young mice further corroborate with lower protection in the aged group. It is worth mentioning here that apart from the CD4^+^ Th1 and Th2 cells, age-associated CD4^+^ Treg cell accumulation is likely to play a major role in the increased severity during *L*. *major* infection in old mice [7]. Additionally, it has also been hypothesized further that manipulation of Treg activity may enhance immune responses in the aged population and may therefore be envisioned to improve the vaccine efficiency in aged population. Future studies are needed to elucidate the specific role of Treg cell population in *LdCen-/-* vaccine induced immunity in aged mice.

Overall the outcome of the above described mechanism of immune responses induced by live attenuated *LdCen-/-* parasites resulted in significantly reduced parasite burden in the visceral organs of aged mice compared to naive challenged controls. Thus, *LdCen-/-* parasites are capable of inducing protective immune response in senescent mice in spite of the various deficiencies which have been reported to take place in the general immune response during aging [15]. Finally, to our knowledge this is the first report on evaluation of a genetically modified live attenuated *Leishmania* vaccine in aged mice. It is worth mentioning here, the main reason for using intravenous immunization in the current study with aged animals was to be consistent with our previous studies where all the end points of vaccine induced immunity were derived by intravenous route of immunization in young mice [40]. Nevertheless, in more recent studies using dogs and hamster models, we have shown *LdCen-/-* parasites injected either by subcutaneous (dogs) [42] or intradermal (hamsters) [41] routes also induced a strong protective immunity against *L*. *donovani* infection. We are currently performing a comparative analysis of optimal immune responses of *LdCen-/-* parasites inoculated through different routes of immunization in a mouse model.

In summary, our results thus indicate that *LdCen-/-* immunization induces substantial protection in aged host against virulent *L*. *donovani* challenge via induction of heightened innate effector function and subsequent predominant Th1 response. However, due to immunosenescence the adaptive immune responses are lower in aged mice compared to young and attempts could be made in future in improving this vaccine efficacy in aged host via administration of either higher vaccine dose, changing the vaccination route (intradermal) or addition of adjuvants such as sand fly salivary gland proteins. In that regard, a recent study from our group has shown induction of a long-lasting protective immune response in young hamsters immunized intradermally with salivary protein LJM19 with *LdCen-/-* against challenge from virulent parasites [41]. Whether such strategy will work in aged animals remains to be seen and is part of future studies. Taken together, our studies suggest that live attenuated *LdCen-/-* vaccine candidate has the potential to be used across all age groups against VL.

## Supporting Information

S1 Fig Dendritic cells sort selected from *LdCen-/-* infected young and aged mice are more efficient antigen presenting cells than those from *LdWT* infected mice. Parasitized DCs were sorted from different groups of young and aged mice after 4 days of infection. DCs were pulsed with OVA peptide and then cocultured with purified CFSE-labeled CD4+T cells from DO11.10 transgenic young and aged mice to measure T cell proliferation and cytokine (IFN-γ and IL-10) production by flow cytometry. (A) Gating strategy used to study T cell proliferation and cytokine production. (B) Cumulative data representing T cell proliferation of CD4+ T cells. Cell proliferation was done in triplicates and represented by the bar diagram. (C) The ratio of IFN-γ to IL-10 produced from proliferated CD4+T cells. The data presented are representative of three experiments with similar results. Mean and SEM of six mice in each group are shown. *p < 0.*05*, **p < 0.*005*. Black bar indicates young mice and white bar indicates aged mice.
(TIF)

S2 Fig RT-PCR analysis of the cytokine profiles in splenocytes from naive, *LdCen-/-* immunized (Imm), naive challenged (Naive Chal), and *LdCen-/-* immunized challenged (Imm Chal) young and aged mice. Expression levels of IFN-γ (A), IL-12 (B), TNF (C), IL-10 (D) and IL-4 (E) were measured by RT-PCR analysis after extracting RNA from different groups of naïve, immunized and immunized challenged young and aged mice splenocytes. The data presented are representative of two independent experiments with similar results (n = 6). Mean and SEM of each group are shown. *p<0.*05*, **p < 0.*005*. Black bar indicates young mice and white bar indicates aged mice.
(TIF)
