# Supplementary figures and images for: Live Attenuated Leishmania donovani Centrin Knock Out Parasites Generate Non-inferior Protective Immune Response in Aged Mice against Visceral Leishmaniasis
Source: PLoS Negl Trop Dis. 2016 Aug 31;10(8):e0004963. doi: 10.1371/journal.pntd.0004963 (PMC5007048; doi:10.1371/journal.pntd.0004963)

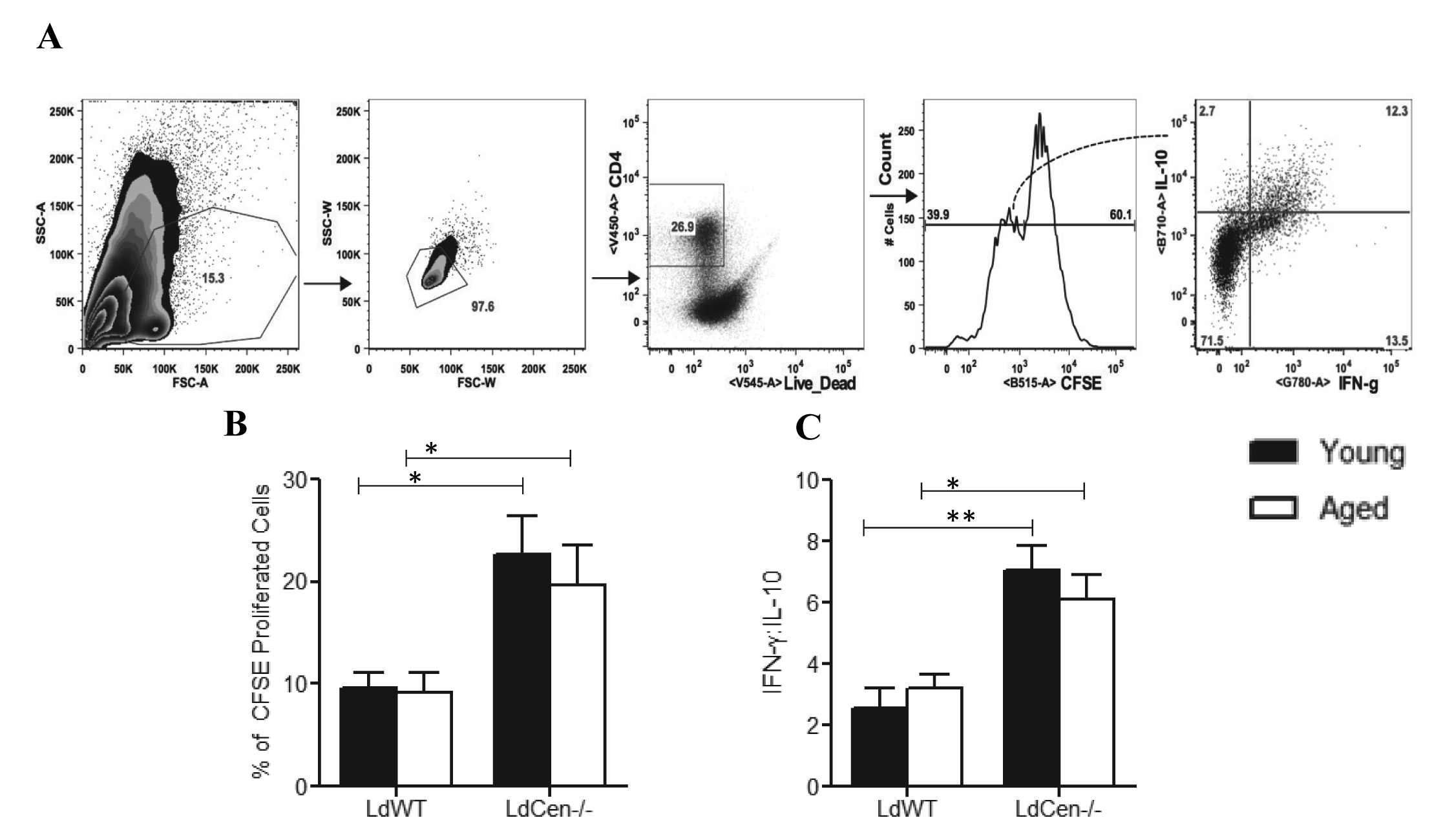

Supplement: S1 Fig — Parasitized DCs were sorted from different groups of young and aged mice after 4 days of infection. DCs were pulsed with OVA peptide and then cocultured with purified CFSE-labeled CD4+T cells from DO11.10 transgenic young and aged mice to measure T cell proliferation and cytokine (IFN-γ and IL-10) production by flow cytometry. (A) Gating strategy used to study T cell proliferation and cytokine production. (B) Cumulative data representing T cell proliferation of CD4+ T cells. Cell proliferation was done in triplicates and represented by the bar diagram. (C) The ratio of IFN-γ to IL-10 produced from proliferated CD4+T cells. The data presented are representative of three experiments with similar results. Mean and SEM of six mice in each group are shown. *p < 0.05, **p < 0.005. Black bar indicates young mice and white bar indicates aged mice. (TIF) [file pntd.0004963.s001.tif]

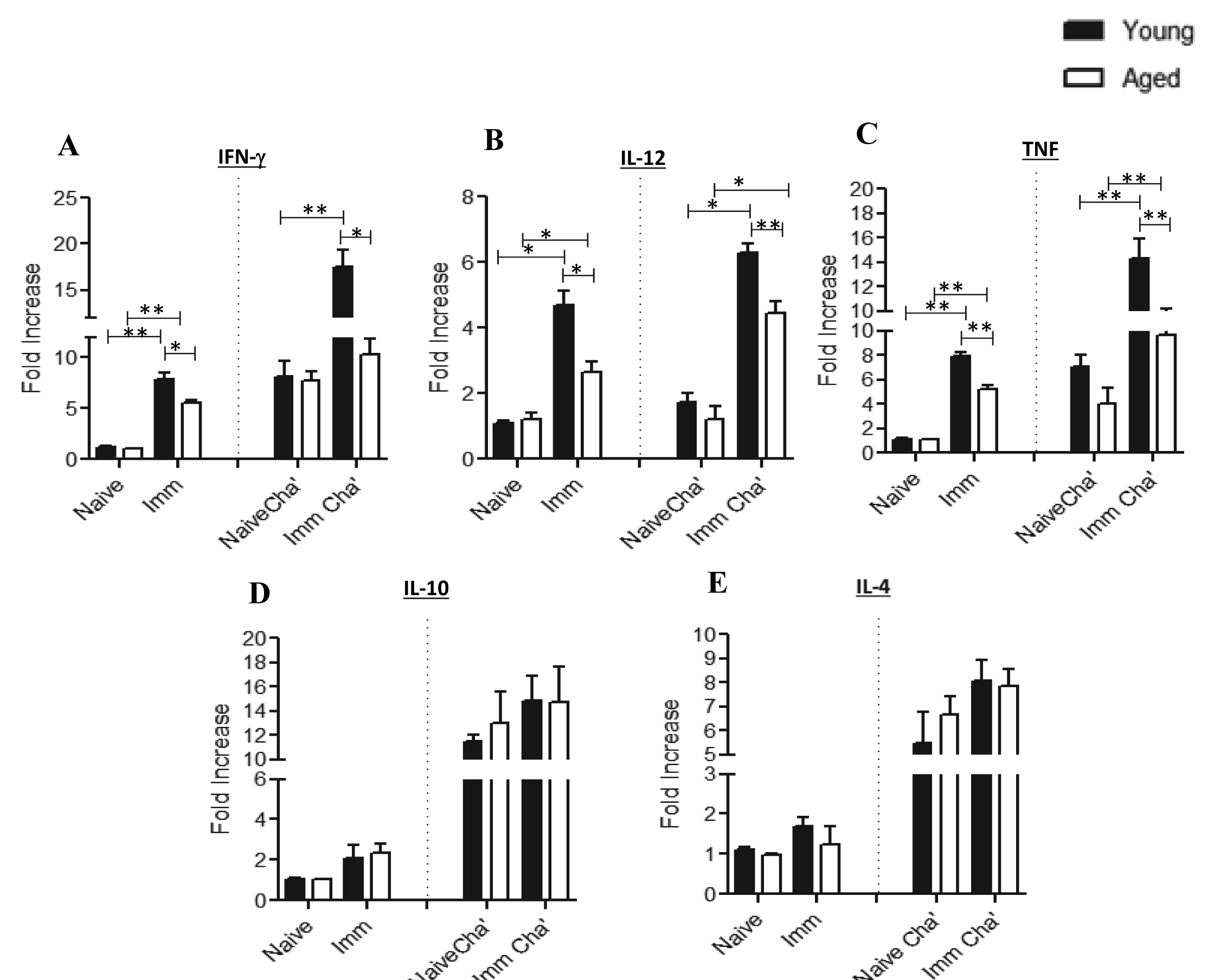

Supplement: S2 Fig — Expression levels of IFN-γ (A), IL-12 (B), TNF (C), IL-10 (D) and IL-4 (E) were measured by RT-PCR analysis after extracting RNA from different groups of naïve, immunized and immunized challenged young and aged mice splenocytes. The data presented are representative of two independent experiments with similar results (n = 6). Mean and SEM of each group are shown. *p<0.05, **p < 0.005. Black bar indicates young mice and white bar indicates aged mice. (TIF) [file pntd.0004963.s002.tif]
